# The Impact of Sulfur-Containing Inorganic Compounds during the Depolymerization of Lignin by Hydrothermal Liquefaction of Black Liquor

**DOI:** 10.1021/acs.energyfuels.3c04737

**Published:** 2024-03-19

**Authors:** Maximilian Wörner, Lukas Werner, Ursel Hornung, Nicholas Islongo Canabarro, David Baudouin, Nicolaus Dahmen

**Affiliations:** † Institute of Catalysis Research and Development (IKFT), Karlsruhe Institute of Technology (KIT), Hermann-von-Helmholtz-Platz 1, Eggenstein-Leopoldshafen 76344, Germany; ‡ Bioenergy and Catalysis Laboratory (LBK), Paul-Scherrer-Institute (PSI), Forschungsstrasse 111, Villigen 5232, Switzerland

## Abstract

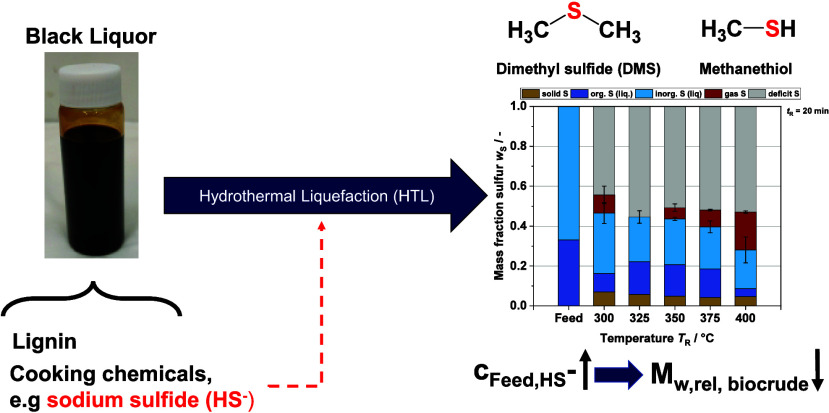

Lignin is a promising resource for the sustainable production of platform chemicals and biofuels. The paper industry produces large quantities of lignin every year, mostly dissolved in a black liquor. With the help of hydrothermal liquefaction, black liquor can be used directly as a feedstock to depolymerize the lignin to desired products. However, because various cooking chemicals (e.g., NaHS, NaOH) used in the Kraft process, dominant in the paper industry, are also dissolved in the black liquor, it is necessary to study in detail their influence on the process as well as their fate. In this work, the focus was on the fate of sulfur and the influence of sulfide (HS^–^). For this purpose, hydrothermal liquefaction experiments (250–400 °C) were carried out with black liquor and self-prepared model black liquor with different sulfide concentrations (0–3 g·L^–1^ HS^–^) in batch reactors (*V* = 25 mL), and the products were analyzed to understand the chemical pathways involving sulfur. It was found that the inorganic sulfur compounds react with organic matter to produce organic sulfur compounds. Dimethyl sulfide is the most abundant of these products. The HS^–^ concentration correlates with the amount of dimethyl sulfide produced. Because methanethiol has also been qualitatively detected, the reaction mechanism of Karnofski et al. for the formation of dimethyl sulfide in the Kraft process also applies to the hydrothermal liquefaction of black liquor. Increased sulfide concentration in the feed leads to an accelerated depolymerization of lignin. In contrast, the yields of some aromatic monomers decrease slightly, possibly as a result of repolymerization reactions also occurring more quickly.

## Introduction

Climate change is increasingly impacting the human quality of life. To mitigate these effects, it is necessary to reduce greenhouse gas (GHG) emissions. This means replacing more and more fossil resources with renewables in the coming years to accomplish the set climate goals. In 2022, the global primary energy demand was at 604.04 EJ, of which 494.05 EJ originated from fossil resources.^1^ These figures show that there is great potential for drastically reducing global warming. One part of the solution is to change carbon feedstocks from fossil resources such as crude oil or coal to biomass that does not compete with food production. The final scenario would be a complete change of concept from a traditional refinery to a biorefinery that delivers industry and energy sectors with biobased fuels and chemicals.^2^ Many different biomass species can be used for this propose, the most abundant one being lignocellulosic biomass.^3^ Each year, 181.5 billion tons are produced from agricultural, grass, or forest land, and only 8.2 billion tons of this is currently being used.^4^ However, the use of these feedstocks involves many challenges, which still need to be overcome. The main reason for this is the complexity of the biomass composition. It is important to know how the feedstock in a conversion process behaves to create a whole biorefinery concept around it.

**Figure 1:**
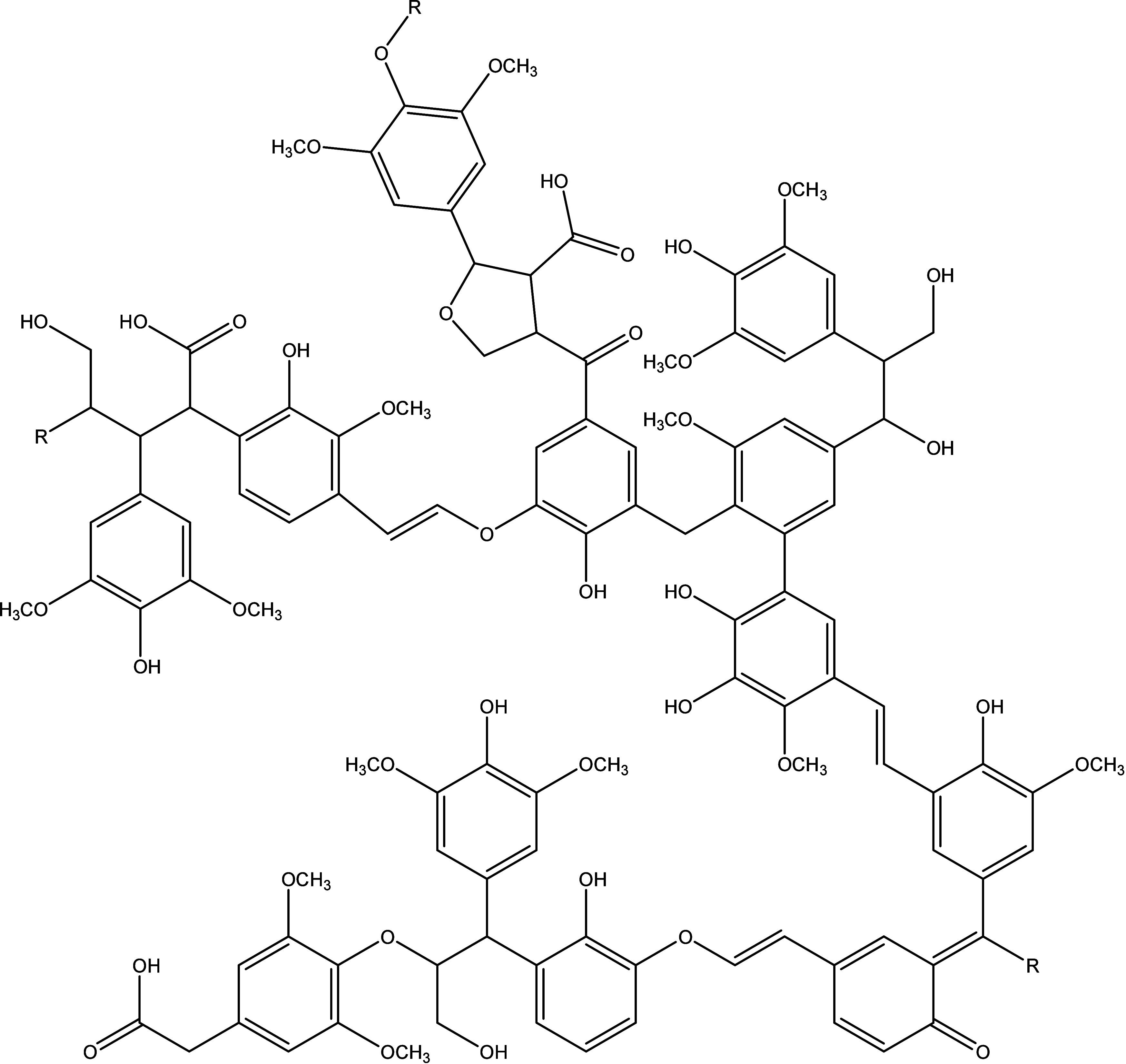
Cutout of a possible lignin structure produced with permission from ref (8). Copyright 2013 Elsevier.

One material with great potential to impact the energy sector from the bottom up is lignin. It is a biopolymer found in every lignocellulosic plant in the world as a stabilizer in cellular walls, conferring structural integrity to plants.^5,6^ It is also the only naturally occurring molecule with a high density of aromatic rings (Figure 1). The macromolecule is built up from three different phenyl propanoids: coniferyl alcohol, sinapyl alcohol, and *p*-cumaryl alcohol. These three molecules are bound together via various bindings, leading to a highly branched macromolecule. In nature, lignin is formed via biosynthesis with the help of different enzymes.^7^

Because lignin is already available in large quantities today and does not negatively affect the food versus fuel debate, it has received increasing focus in recent years as a possible biofeedstock for a potential biorefinery.^4,9^ The possibilities offered by lignin in the context of a chemical conversion are manifold. The most important among the few commercial uses of lignin in a chemical process to date is the production of vanillin.^10^

A major producer of lignin, albeit only as a byproduct, is the paper industry. A total of 50 million tons are produced annually in this sector.^11^ The cellulose fibers are separated from the remains of the wood using various processes, of which the Kraft process is the most frequently used in pulp mills worldwide. Most of the lignin is burned to generate electricity and heat as well as to recover pulping chemicals. The energy thus generated is conventionally used to cover the pulp mill’s energy consumption. However, large and modern pulp mills generate a significant surplus of energy, which is sold on the market.^12^ In addition, a large amount of energy is required to evaporate water and concentrate black liquor from 15 to 20 wt % up to 65–80 wt %.^13^ Alternatively, the surplus lignin could be recovered for chemical use, i.e., to process the lignin into products of higher added value compared to electricity generation.^14^

**Figure 2:**
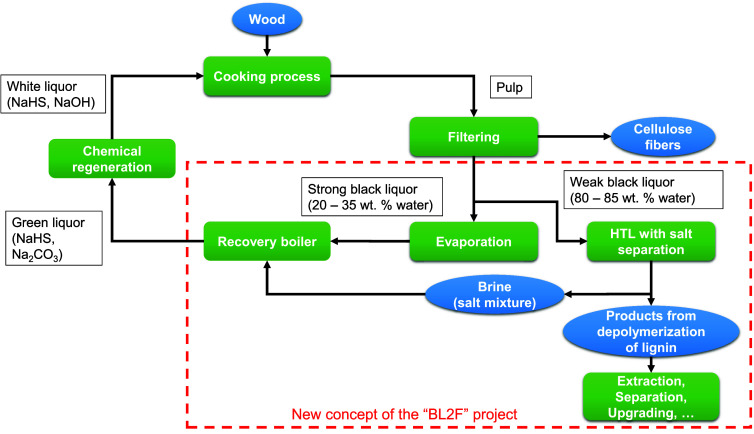
Kraft process with integrated HTL with salt separation, valorizing a part of the BL stream to chemicals.

To convert the lignin into useful products, a broad range of processes appear applicable. Possible conversion methods are biochemical,^15^ electrochemical,^16^ catalytic,^17^ and thermochemical processes.^18^ Of the latter, pyrolysis^19,20^ and hydrothermal conversion^21^ are the most relevant. For hydrothermal conversion to fuels or useful compounds such as aromatics, hydrothermal liquefaction (HTL)^22−24^ is considered the most promising option. Compared to pyrolysis, HTL has the advantage that wet biomass can be used directly without prior drying. Because lignin is primarily dissolved in the black liquor (BL), it is possible to save the drying step and therefore energy when using the HTL process. The EU-Project “Black Liquor to Fuels” (BL2F) investigates how HTL can be integrated into a pulp mill (integrated HTL) (see Figure 2). The conventional Kraft process makes use of a recovery boiler to burn the black liquor that is first concentrated using multieffect evaporators. The idea behind the BL2F project is to integrate an HTL plant with salt separation. The raw BL is fed directly into the HTL reactor, and the top product, called desalinated stream, can be processed further.^25,26^ The brine (bottom stream), containing most of the cooking chemicals, is recovered and fed back into the Kraft process. Note that the efficiency of the separation of these salts strongly depends on the temperature, which should ideally be above the critical point of the water.

In HTL, the changing properties of water close to the critical point (*T*_c_ = 374 °C, *p*_c_ = 221 bar) are utilized to enable depolymerization of the lignin molecule dissolved in the water. In this study, reaction temperatures between *T*_R_ = 300 and 400 °C are investigated in batch mode at pressures between *p*_R_ = 200 and 300 bar. Under these process conditions, water serves as a reaction medium, reactant, and, at the same time, acid catalyst.^27^ Because of the high ionic product of water, *K*_w_, a high H^+^ and OH^–^ concentration is established in the reaction medium, up to a factor of 1000 larger than that at ambient conditions. As a result, many acid- or base-catalyzed reactions are accelerated.^28,29^ Another positive effect is the significantly improved solubility of organic compounds in near-critical water due to the reduced relative permittivity. Consequently, the desired reactions can occur faster and more effectively.

There are several studies on hydrothermal liquefaction of lignin and model substances, which describe the process and investigate specific parameters.^13,21,30^ Some are highlighted in the following. Studies by Belkheiri et al. investigated the HTL of lignin at different pH values.^31^ It is shown that a higher pH value leads to more water-soluble organic products, whereas the influence on the biocrude fraction was not clearly determinable. Further studies on the HTL of lignin deal with the use of catalysts. Forchheim et al., for example, have shown in their work that Raney nickel had no influence on the depolymerization of lignin but catalyzed the hydrodeoxygenation (HDO).^32^ This results in higher gas yields as well as higher phenol concentrations in the product. The most commonly investigated type of lignin derives from the Kraft process and is soluble primarily in alkaline environments. Alkali metal salts are often already present in the feedstock and bring catalytic effects in addition to improved solubility of the Kraft lignin, which has a good solubility at high pH. Mostly sodium and potassium salts (NaOH/Na_2_CO_3_, KOH/K_2_CO_3_) are used. Belkheiri et al. showed that a shift from potassium to sodium salts only has a minor effect on the product phase distribution.^54^ The work of Rana et al.^33^ investigated several salts of both basic and acidic nature, for example, K_2_CO_3_ or AlK(SO_4_)_2_·12H_2_O, and it was found that K_2_CO_3_ provides the best results in terms of biocrude yields as well as low char formation. Forchheim et al. were able to propose a compact reaction network based on their HTL experiments with lignin and various model substances such as guaiacol.^34^ The work indicates that catechols and phenols are the main products among the monomers formed. A common feature of most experiments performed with lignin to date is that the lignin was recovered from the pulp solution by precipitation beforehand and then acidified and dried. In contrast, Orebom et al.^55^ used BL as feedstock in HTL in batch experiments. They found that maximum biocrude yields are achieved between a reaction temperature *T*_R_ of about 370 and 380 °C. They also showed that the best results were achieved with a dry matter content of 16 wt %. They investigated the reaction temperature range from 340 to 420 °C and the dry matter content range between 16 wt % and approximately 60 wt %.

One issue that has not been addressed in the study by Orebom et al. is the role of the various cooking chemicals that find their way into the BL in the Kraft process. It should be noted that the pulp industries strive to recycle these salts (see the Kraft process diagram). In an integrated HTL of the BL, as studied in the BL2F project, this recycling process must therefore also be implemented. Wang et al.^25^ have studied this topic in their work. They were able to collect up to 96% of the salt in the wastewater stream. However, their study was performed without organic compounds, and it is important to explore the influences of the salts on the HTL of lignin. Indeed, it can be expected that product distribution and composition are influenced by the salts. The majority of these salts are sodium carbonate (Na_2_CO_3_), sodium sulfate (Na_2_SO_4_), and sodium hydrosulfide (NaSH). Table 1 lists the mass fraction of inorganic compounds in a typical BL based on dry matter analysis from Niemelä and Alén.^35^ The high carbonate content originates from Kraft process reactions. In a conventional process, this carbonate is converted to NaOH again in the causticization step. The other salts contain mainly potassium or chlorine.

**Table 1:** Mass Fraction of Inorganic Salts in BL Based on Pine Wood after the Kraft Process (Dry Matter Based)^35^

**Na**_**2**_**S**	**Na**_**2**_**SO**_**4**_	**Na**_**2**_**S**_**2**_**O**_**3**_	**Na**_**2**_**SO**_**3**_	**NaOH**	**Na**_**2**_**CO**_**3**_	**others**
17 wt %	12 wt %	14 wt %	7 wt %	6 wt %	32 wt %	12 wt %

Although some of these salts are desired or even required in the feedstock for HTL, as already described above, this does not apply to the salts that contain sulfur. The inorganic sulfur compounds are present as anions in the BL, mainly as bisulfide (HS^–^),^36^ sulfite (SO_3_^2–^), sulfate (SO_4_^2–^), and thiosulfate (S_2_O_3_^2–^). In this context, bisulfide plays an important role because it is very corrosive to metals and alloys. Furthermore, it is in chemical equilibrium with hydrogen sulfide, which is toxic, in addition to the unpleasant odor in the smallest quantities. A further aspect is the formation of organic sulfur compounds. Here, volatile molecules such as simple organic (poly)sulfides (R_1_-(S)_*x*_-R_2_) or thiols (R-SH) play a major role. Some of these compounds, just like hydrogen sulfide, are very toxic and extremely dangerous for aquatic organisms due to their good water solubility.^37^ For this reason, the emissions of these substances must be monitored and kept at low levels. From an engineering perspective, the concentrations of sulfur compounds before and after the HTL must also be known for proper process design. This relates to both corrosion resistance and the use of catalysts. Because sulfur and its compounds are very strong catalyst poisons,^38^ resistant catalysts must be used accordingly for biocrude upgrading steps. If this is not technically possible or if fuels are the desired end product, hydrodesulfurization (HDS) must be incorporated into the process chain.^39^ Finally, the formation of thiols, having a p*K*_a_ in the range 10–11, might lead to sodium losses in the context of HTL integration into a Kraft process, which is not desired.

As such, the fate of sulfur during the HTL needs to be studied. A first indication of what happens to the inorganic sulfur compounds in interaction with lignin under hydrothermal conditions is provided by looking at the Kraft process itself, where hydrothermal conditions already prevail.^40^ For example, Karnofski et al. have illustrated the reaction of the HS^–^-ion with the methoxy groups of lignin. In this process, dimethyl sulfide (H_3_C-S-CH_3_, DMS) is formed via the intermediate product methanethiol (H_3_C-SH) (see eqs 1–3).^41^ Bordado and Gomes characterized DMS and methanethiol as two of the main sulfurous byproducts in the flue gas of Portuguese pulp mills.^42,43^
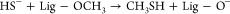
1
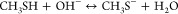
2
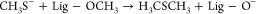
3

Regarding the issues explained above, there are unsolved questions about the role of sulfur during the HTL of lignin using BL directly as a feedstock. Where does sulfur end up after the process? What kind of organic or inorganic sulfur compounds are formed? Do the sulfurous salts have any influence on the HTL process, especially the sulfide? To tackle these questions, we analyzed all product phases produced from the HTL of BL obtained at different reaction temperatures (*T*_R_'s) with a special focus on the sulfur content and its chemical nature via different analytical methods. We paid special attention to the gas and liquid phases to characterize and quantify organic sulfur compounds. Another point of interest was the influence of the different sulfur anions on the HTL process. We expected that these anions, especially HS^–^, could accelerate the depolymerization process because it is also used in the Kraft process for cleaving the ether bonds. As such, batch experiments with model black liquors (MBLs) containing different concentrations of HS^–^ anions were also performed. Another point dealt with in this work is the influence of the HS^–^ concentration on the yields of aromatic monomer compounds, which tend to be the main product of the HTL of lignin. Furthermore, the correlation between the HS^–^ concentration and specific sulfur compounds was investigated. To accomplish these goals, we performed various analysis methods focusing on sulfur and specific sulfur compounds. The influence of the sulfide concentration was investigated by measuring the changes in yields of specific aromatic product compounds and the relative molecular mass of the produced biocrude.

## Materials and Methods

### Feedstocks Used for the HTL

The black liquor used in our experiments was delivered from a Figueira da Foz pulp mill (The Navigator Company) in Portugal. It originates from eucalyptus wood. The BL comes as a black, almost homogeneous liquid. The properties of the BL are listed in Table 2. *W*_tr_ is the dry matter of the BL after 24 h at 105 °C, *w*_ash,815 °C_ is the remaining ash after 4 h at 815 °C, and *w*_BL, burnable_ is the burnable fraction of the BL calculated from the loss on ignition from the dry matter. The yields calculated in the results section are based on *w*_BL, burnable_, as we assume that the burnable fraction is close to the organic fraction (see Cardoso et al.^44^). Differences may arise due to the change in the ash composition as a result of combustion. The strong alkaline salt concentration results in pH over 12.5. The feedstock was stored in a refrigerator at 5 °C to slow possible oxidation processes. Freezing was not applicable due to possible mechanical destruction of the lignin molecules. The mass fractions of elements found in the dry matter are listed in Table 3. They are in line with typical BL from hardwood like eucalyptus used in a Kraft process and fit well into the values found by Cardoso et al.,^44^ who compared the chemical composition of different BL from different pulp mills. The mass fractions of extracted lignin from BL are also listed in Table 3.

**Table 2:** Properties of the BL Used in the Experiments^a^

**dry matter,***w*_tr_	**ash content**, *w*_ash*,***815** °**C**_	**dry matter-based loss on ignition**	**raw BL-based burnable matter,***w*_**BL, burnable**_	**density, ρ**_BL_	**pH**
14.5 wt %	6.1 wt %	57.9 wt %	8.4 wt %	1.0725 kg·L^–1^	>12.5

a *w*_BL, burnable_ was calculated from the loss on ignition corrected from the dry matter.

**Table 3:** Elemental Composition of the Dry Mass of the BL and of the Extracted Lignin^a^

**element symbol**	**mass fraction dry mass** BL/wt %	**mass fraction extracted** lignin/wt %
C (EA)	34 ± 0.4	60.3 ± 0.1
H (EA)	3.4 ± 0.5	5.7 ± 0.1
N (EA)	<0.1	<0.1
S (EA)	4.7 ± 0.1	2.6 ± 0.1
O (diff.)	38.8	31
Na (ICP)	17.7 ± 0.9	0.4 ± 0.02
K (ICP)	1.3 ± 0.06	<1
sum	100	100

a Analysis was performed via elemental analysis (EA) and inductively coupled plasma-optical emission spectrometry (ICP-OES); oxygen was calculated via difference; no other element was detected in relevant amounts.

Model black liquor (MBL) consisting of lignin and sulfur chemicals is prepared to adjust the HS^–^ concentration as accurately as possible. The composition of this model mixture is based on the characterization of the real BL. To get as close as possible to the original, the lignin extracted from the BL using the LignoBoost process^45^ is used. The salts required for the liquor are sodium sulfide (Na_2_S, in the form of Na_2_S nonahydrate), sodium sulfite (Na_2_SO_3_), sodium thiosulfate (Na_2_S_2_O_3_), sodium sulfate (Na_2_SO_4_), sodium and potassium carbonate (Na_2_CO_3_/K_2_CO_3_), and sodium and potassium hydroxide (NaOH/KOH). Four model liquors with 0, 1, 2, and 3 g·L^–1^ HS^–^ (models A–D) are prepared. Model D is the one closest to the real BL sample. The measured HS^–^ concentration in the BL is between 3 and 3.5 g·L^–1^. The HS^–^ concentration is varied by adding Na_2_S. To neglect possible effects due to higher sodium concentrations, the other salt concentrations were also adjusted. Table S1 in Supporting Information lists the weighed-in masses of the salts used and the volume of NaOH/KOH solution for pH adjustment for the four model liquors. The potassium carbonate is also changing because we wanted to keep the Na/K ratio similar for each MBL. The NaOH/KOH solution used contained around 20 wt % NaOH and 1.5 wt % KOH and was added until a pH of approximately 12.5 was achieved.

### Batch Experiment Setup and Product Separation

The batch experiments were performed in micro autoclaves (*V* = 25 mL) made of stainless steel 1.4571 (316Ti). In the first step, a certain amount of the BL was filled into the micro autoclaves. Then an inert atmosphere was created with N_2_. At an initial pressure of 10 bar, the reactor was sealed. The amount of BL in the reactor varied depending on the desired reaction temperature. Because of the change in the density of water at different temperatures and the corresponding change in pressure, it was necessary to compensate for this effect by adjusting the feedstock volume. The different volumes are given with the respective reaction temperature in Table S2 in the Supporting Information (SI). The feedstock volumes were estimated from the density of pure water at each temperature.^46^ The pressure was then set around 200 to 250 bar. The heating process was carried out in a fluidized sand bath (SBL 2, Techne, Stone, UK). A preheating time *t*_pre_ = 10 min was implemented, which was confirmed to be sufficient to reach the desired reaction temperatures. Reaction temperatures for HTL with real BL were set between *T*_R_ = 250 and 400 °C with a holding time of *t*_R_ = 20 min (overall *t* = *t*_R_ + *t*_pre_). HTL with the MBL was carried out at *T*_R_ = 375 °C and *t*_R_ = 10 min. After the HTL process, the autoclaves were immediately cooled in a water bath to interrupt the ongoing reactions. After taking a gas sample with a gastight syringe, the reactor was removed from the setup, and the solid product was separated from the liquid phase by vacuum filtration. The nylon filter used has a diameter of 47 mm and a pore size of 0.45 μm (Whatman, GE Healthcare, Buckinghamshire, UK). The solid residue was dried at *T* = 105 °C for 24 h. Liquid–liquid extraction (LLE) was performed to separate the organic phase from the aqueous phase. Two milliliters of the liquid phase had to be acidified with 6 M hydrochloric acid to a pH of 2–4. After filtration, 0.52 mL of ethyl acetate was added to 1.3 mL of the filtrate, which served as the extractant. After shaking, the sample was allowed to rest in the vial for 1 h to allow for complete phase separation.

### Analytical Procedure and Assessment

The gas sample was analyzed by gas chromatography (GC 6890N, GC 7890B, Agilent, Santa Clara, CA, USA). The detectors used were a flame ionization detector (for DMS), a temperature conductivity detector (for H_2_S), and a mass spectrometer detector (5973 MSD, Agilent, Santa Clara, CA, USA). In both, a column specific to the analysis of sulfur compounds was used (RT-U Bond 15 m, 0.25 mm, 0.25 μm, Restek, Bellefonte, PA, USA). For GC-FID/TCD, the sample was injected at 150 °C in a 1:2 split mode. The carrier gas was helium (8.69 mL·min^–1^). The heating ramp started at 60 °C with a holding time of 1 min and heated up to 180 °C with 40 °C·min^–1^ and a holding time of 5 min. The sample at GC–MS was injected at 280 °C in splitless mode. Helium was used as carrier gas (1.5 mL·min^–1^). The temperature ramp started at 30 °C for 5 min followed by a heating ramp of 8 °C·min^–1^ until 200 °C, which was held for 5 min. The MSD was operated in 70 eV EI (electron impact ionization) mode with a source temperature of 230 °C and a quadrupole temperature of 150 °C with scanning from 30 to 550 *m*/*z* with a frequency of 4.5 scans per second. The elemental composition of the dried solid residue was determined by elemental analysis (EA; Vario EL cube, Elementar Analysetechnik GmbH, Hanau, Germany) and inductively coupled plasma-optical emission spectrometry (ICP-OES; ICP-725, Agilent Technologies, Santa Clara, CA, USA) after microwave-assisted acid digestion in reverse aqua regia (mixture of conc. HNO_3_ (65 wt %) and conc. HCL 3:1 (37 wt %). An aliquot of the liquid phase before LLE phase was examined by several analytical methods: to determine the inorganic as well as the organic sulfur content, 0.1 mL of the sample was mixed with 0.5 mL hydrogen peroxide, 1 mL potassium hydroxide, as well as 8.4 mL purified water. This step allows all inorganic sulfur to be converted to the highest oxidation state (+VI) in the form of sulfate ions (SO_4_^2–^), as HS^–^ ions strongly influence measurements and are problematic to some instruments. Sulfate can be easily quantified using ion chromatography (IC-An; IC professional detector, 930 compact IC flex, interface 830, 858 professional sample processor, column Metrosep C3–250, Metrohm, Herisau, Switzerland). The molar concentration of sulfate was calculated using the measured mass concentration β_SO_4_^2–^_ and the molecular mass of sulfate *M*_SO_4_^2–^_ (see eq 4). The mass concentration of the inorganic sulfur β_S, inorg_ was obtained using eq 5.^47−49^
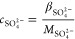
4
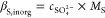
5

In parallel, a second aliquot was analyzed by using elemental analysis (EA) to determine the total sulfur content. The difference between total sulfur and inorganic sulfur was used to determine the organic sulfur content in the liquid phase. A GC with a sulfur chemiluminescence detector (SCD, GC 7890A, 355 SCD, column G3903-63002, Agilent, Santa Clara, CA, USA) was used for the determination and quantification of organic sulfur compounds. The carrier gas was helium (3 mL·min^–1^). The sample was injected at 255 °C in a 1:10 split mode. The oven temperature ramp started at 40 °C and held this temperature for 7 min before it increased at a 7 K·min^–1^ heating rate up to 220 °C for 8 min. For sample preparation, 0.5 mL of the liquid phase was dissolved in 1.5 mL of isopropanol and filtered. Together with the sulfur content in the solid phase and the detected and quantified sulfur compounds in the gas phase, a mass balance of sulfur can be prepared.

The effect of the HS^–^ concentration on the HTL process was determined from the yields of typical aromatic compounds and the molecular weight of the biocrude by using the extracted organic phase. To determine the mass concentration of individual aromatics, β_i, raw_, a GC–MS (GC 6890N and 5973 MSD mass spectrometry detector, Agilent, Santa Clara, CA, USA) and a GC-FID (GC 7820A, Agilent, Santa Clara, CA, USA) were used. In both cases, the Restek RTX-5 column was used (RTX-5, 30 m, 0.32 mm, 0.5 μm, Restek, Bellefonte, PA, USA). For GC–MS, the sample was injected at 280 °C in splitless mode. Helium was used as carrier gas (1.5 mL·min^–1^). The temperature ramp of the GC–MS oven started at 35 °C for 5 min followed by a heating ramp of 8 °C·min^–1^ up to 240 °C, which was held for 10 min. The MSD was operated in 70 eV EI (electron impact ionization) mode with a source temperature of 230 °C, a quadrupole temperature of 150 °C, and a solvent delay of 4.5 min followed by scanning from 35 to 350 *m*/*z* with a frequency of 4.5 scans per second. For GC-FID, the sample was injected at 280 °C in a 1:50 split mode. The carrier gas was helium (1.2 mL·min^–1^). The heating ramp started at 50 °C with a holding time of 2 min, heating up to 190 °C with 8 °C·min^–1^ in the first step and up to 230 °C with 20 °C·min^–1^ in the second step, and a holding time of 5 min. For quantification, we used pentadecane as an internal standard (ISTD). Together with the distribution coefficients *K*_i_'s (see Table S3 in Supporting Information) for the individual substances, which describe the distribution of a specific compound in a mixture of ethyl acetate and water, it was possible to calculate the concentration in the total liquid phase β_i_ (see eq 6). Factor *a* is the total dilution of the original sample, *b* takes into account the ratio between the volume of the sample and the ethyl acetate, and *c* is the ISTD factor. The yield *Y*_i, BM_ related to the biomass used in the feedstock can be calculated using eq 7 with the obtained mass for the liquid product *m*_liq, prod_ and the mass of feedstock used *m*_feed_.
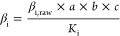
6
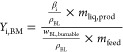
7

Only the three main components catechol, 3-methylcatechol, and 4-methylcatechol were considered. In addition, the yields of di- and trimethylcatechols were semiquantified via peak area equivalents.

To determine the molecular weight of the feedstock lignin and the produced biocrude, the ethyl acetate must be evaporated after the extraction step. Then a spatula tip of the biocrude was dissolved in 2–3 mL of dimethyl sulfoxide (DMSO). The molecular weight was determined by size exclusion chromatography (SEC; LaChrom diode array detector DAD L-2455, Merck, Darmstadt, Germany, with a Viscotek A2500 column, Malvern Panalytical, Malvern, UK). An exact determination of the molecular weight is not possible because possible interactions are taking place between the sample column and solvent and cannot be distinguished.

All experiments were repeated three times. Most of the analysis was performed once for each sample. A mean value was calculated from the three repetitions along with the standard deviation. The SEC analysis and the sodium and potassium balance mixtures of the three samples were used for the elemental analysis.

## Results and Discussion

### Effect of Reaction Temperature *T*_R_ on the Sulfur Mass Balance

**Figure 3:**
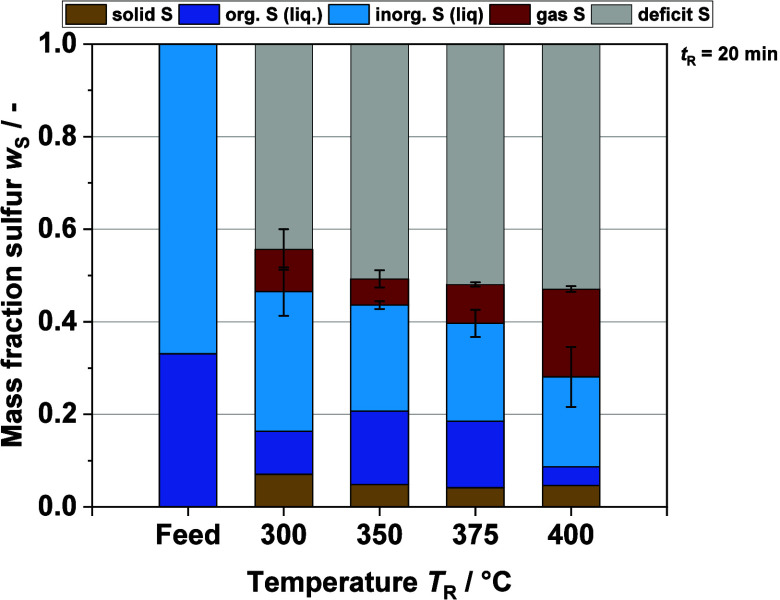
Sulfur mass balance at different reaction temperatures *T*_R_'s and in the BL feedstock.

Figure 3 shows the sulfur mass balance for *T*_R_ = 300–400 °C and *t*_R_ = 20 min. The mass fractions are separated into five different sections: total sulfur in the solid phase, organic and inorganic sulfur in the liquid, sulfur in the gas phase, and the sulfur deficit in the balance. In addition, the organic and inorganic sulfur in the liquid BL feed is given in the diagram as a reference. For all the investigated samples from the HTL of BL, we were able to detect around 50 wt % of all sulfur in the system. The high deficits are probably mainly due to two reasons. First, we can only quantify two gas components, hydrogen sulfide (H_2_S) and DMS, whereas a quantitative determination of methanethiol, the intermediate product according to Karnofski et al. (see eq 2), and other gaseous sulfurous components is not possible with the analysis setup used in this work. Also, we can only quantify one part of the H_2_S because the pH after reaction is between 8.5 and 10 (see Figures S1 and S2 in the Supporting Information) compared to the p*K*_a_ of H_2_S, which is 7. This leads to an underestimation of the level of sulfur in the gaseous phase. Second, thiols and small sulfides are very volatile components (for example, DMS *T*_boil_ = 34 °C, methanethiol *T*_boil_ = 6 °C) and can lead to an underestimation of sulfur recovery in the organic sulfur mass fraction of the liquid phase, particularly after a decrease of the pH. Note that shifts in thermodynamic equilibria are expected when decreasing the pH in the liquid phase, e.g., by dilution, which can result in loss in the gas phase. Examples are methanethiol (CH_3_S^–^ to CH_3_SH) and hydrogen sulfide (HS^–^ to H_2_S).

**Figure 4:**
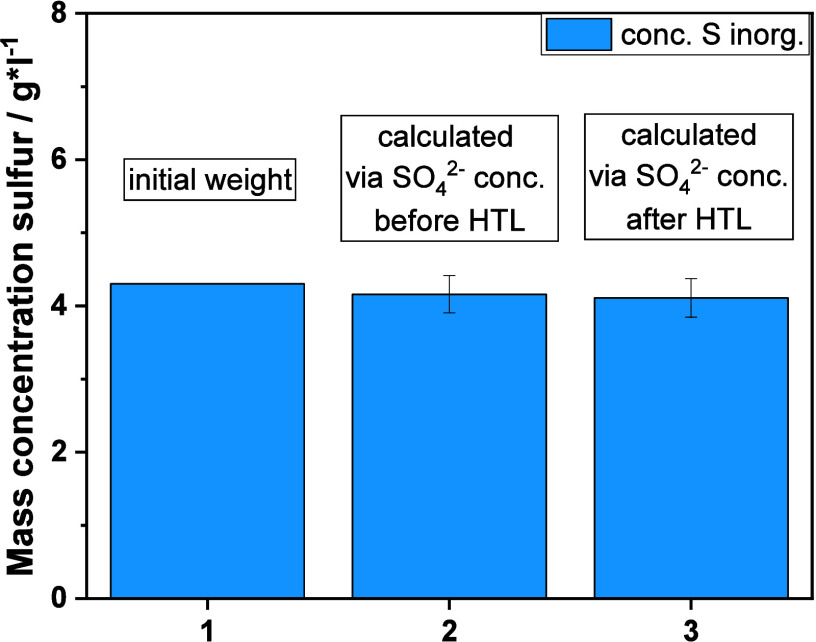
Mass concentration of sulfur for model BL calculated by the weighed masses of salts (bar 1), for model BL without lignin calculated via SO_4_^2–^ concentration determined by IC-An (bar 2), and for the liquid product of HTL of model BL without lignin (*T*_R_ = 375 °C, *t*_R_ = 10 min) calculated via SO_4_^2–^ concentration determined by IC-An (bar 3).

Nevertheless, some important conclusions can be drawn from this sulfur balance, and to our knowledge, it is the best available sulfur balance from HTL of BL to date. The most important statement pertains to inorganic sulfur in the liquid phase, which originates from the various cooking chemicals of the pulping process. At a reaction temperature of *T*_R_ = 300 °C, more than half of the inorganic sulfur has already disappeared from the liquid. This is followed by a further slight decrease until 400 °C. Therefore, reactions between inorganic sulfur and organic matter are very likely, but it cannot be ruled out that the sulfidation of the walls of the reactor (e.g., to nickel sulfides) also plays a role. To validate our procedure, some tests were performed to validate the total inorganic sulfur quantification using a model BL. Figure 4 shows that an almost identical sulfur concentration is achieved with the proposed IC analysis compared with the theoretical sulfur concentration in the MBL. The theoretical sulfur concentration is based on the weighed-in masses of the salts and the lignin used. It can therefore be assumed that the changes in the inorganic sulfur as found in the mass balance are not seriously influenced by the significant material losses. In addition, the sulfur concentration of a MBL without lignin after the HTL process is shown in the same graph. Here also, hardly any deviation can be observed, which validates that the method is reliable for quantification of inorganic sulfur in the product samples. It also excludes the possibility that the reaction or ab-/adsorption of inorganic sulfur with the walls of the reactor has an impact on the total sulfur balance. Plus, it shows that organic material must be present for the sulfur mass balance to change. This in turn also strengthens the assumption that direct reactions of the sulfur salts with the organic matter are occurring during the HTL.

In contrast to the fraction of inorganic sulfur in the liquid phase, interestingly, hardly any changes were found in the fraction of sulfur bound in the solid phase. One assumption at the start of this work was that salts could precipitate. However, because sodium and potassium (see Figures S3 and S4 in the Supporting Information) are also found almost exclusively in the liquid phase as corresponding cations, the precipitation of salts during the performed HTL experiments plays only a minor role.

### Effect of Reaction Temperature *T*_R_ on Organosulfur Compounds in the Liquid Product Phase

**Figure 5:**
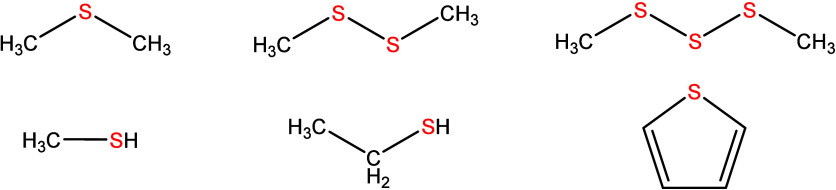
Organosulfur compounds in the liquid phase found by GC-SCD analysis.

A more detailed insight into the chemical nature of the sulfurous components of the liquid phase is possible with GC-SCD analysis, which can identify several typical organosulfur compounds. A majority of the detected compounds are presumably the products of the reaction between the organic material from biomass and the inorganic sulfurous salts (mainly sulfide). These include various sulfides, di- and trisulfides, thiols, and thiophenes (see Figure 5). Quantifiable compounds include DMS, dimethyl disulfide (DMDS), and dimethyl trisulfide (DMTS). Note that DMS can be underestimated because of its low boiling point. All other components could only be detected qualitatively, mostly because their concentrations were too low. Methanethiol is an exception; its chromatogram peaks are the largest after the DMS peaks. However, quantification is not possible because of the low boiling point.

**Figure 6:**
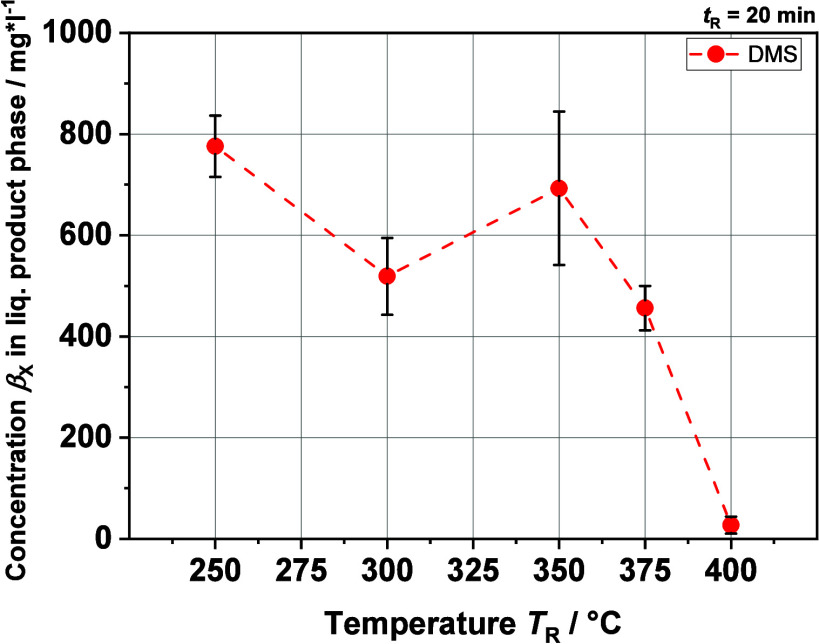
DMS concentration in the liquid product phase from HTL at different reaction temperatures *T*_R_'s generated with GC-SCD.

**Figure 7:**
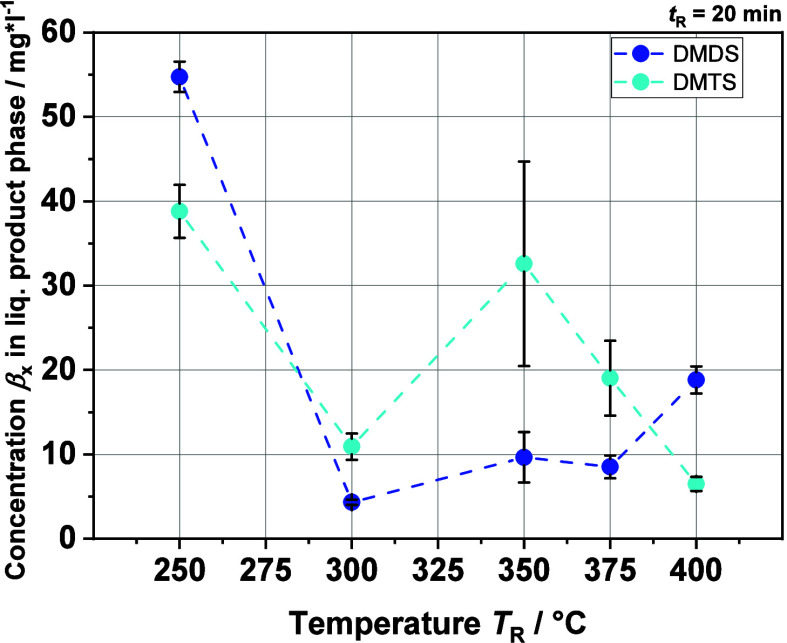
DMDS and DMTS concentration in the liquid product phase from HTL at different reaction temperatures *T*_R_'s generated with GC-SCD.

Figures 6 and 7 show the concentrations of DMS, DMDS, and DMTS in the liquid phase at different temperatures. DMS reaches the highest concentration by far except at *T*_R_ = 400 °C. The trend of DMS concentration correlates to the overall organic sulfur concentration in the liquid phase, showing a general decrease with increasing reaction temperature, which becomes sharp close to *T*_R_ = 400 °C, after which there is a further decrease. This is an indicator that the organic-bound sulfur is mainly present in the form of DMS. The concentrations of the other two quantifiable molecules, DMDS and DMTS, follow the same behavior but at much smaller concentrations by a factor of about 10. This may be due to the (hydro)thermal instability of the S–S bond by homolytic dissociation, whereas in the case of DMS, the C–S bonds need higher temperatures to be cleaved, typically beyond the critical point of water where methyl radical formation is favored.^50^ Note however that disulfides can form from the condensation of two thiols.^51^ In the following course, both concentrations vary between 5 and 20 mg·L^–1^ with the exception of the DMTS concentration at *T*_R_ = 350 °C. At the point mentioned, however, the large error bar must also be taken into account. Why this error is so large at this point for DMS and DMTS in particular cannot be determined.

### Effect of Reaction Temperature *T*_R_ on Sulfurous Compounds in the Gas Phase

**Figure 8:**
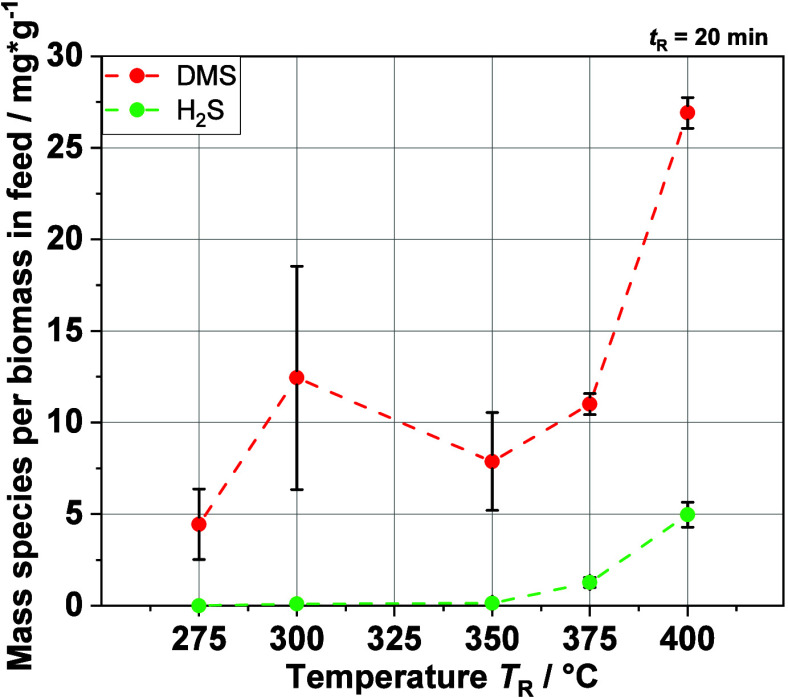
Evolution of the yields of DMS and H_2_S in the analyzed product gas phase after HTL of real BL via GC-FID.

The mass of DMS and H_2_S related to biomass in the feedstock in the gas phase is shown in Figure 8. There is an increase in H_2_S with temperature, but the mass compared to the mass of DMS is very low, as expected because of the high pH of the solution. Below *T*_R_ = 350 °C, no H_2_S is detected, but H_2_S in the gas phase appears at 375 and 400 °C. This can be explained by an equilibrium shift due to a slight decrease in pH with increasing temperature. It also shows that DMS is likely one of the main organosulfur products not only in the liquid phase but also in the gas phase. The increasing masses of both compounds fit very well with the increase in the sulfur content in the gas phase.

**Figure 9:**
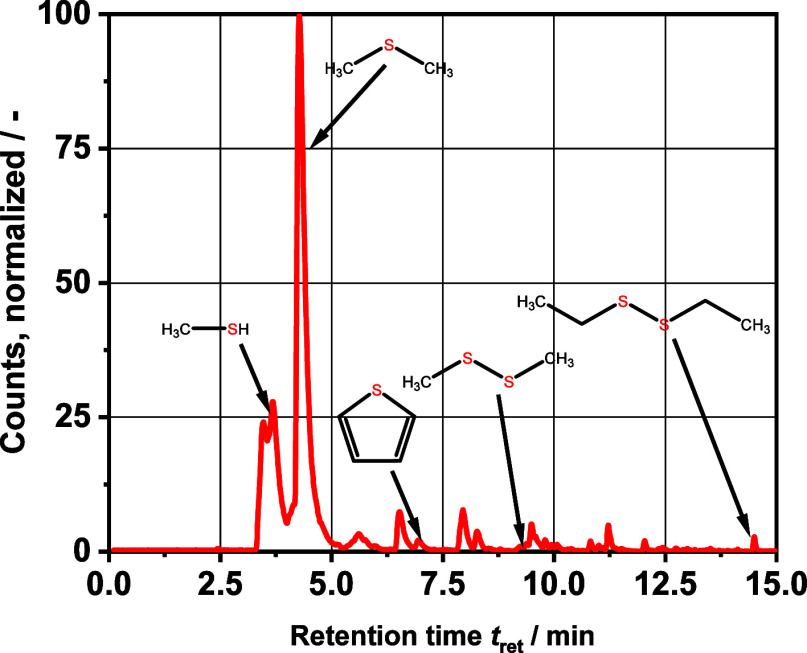
GC–MS chromatogram of a product gas sample; compounds found are methanethiol, DMS, thiophene, DMDS, and diethyl disulfide.

After establishing the sulfur mass balance, we assumed that the largest loss of sulfur occurs via the gas phase. However, with our gas analysis via GC-FID/TCD, we were able to quantify only the two components already shown, DMS and H_2_S. Nevertheless, to find out more about the gas phase, we installed the RT-U Bond column, specially designed for sulfur components, in the GC–MS. The chromatogram of the gas sample can be seen in Figure 9. In agreement with the results discussed above, the DMS peak is by far the largest. Interestingly, it is also possible to detect methanethiol in the gas sample. Its peak area suggests that the sulfur content of methanethiol is of significance in relation to the total mass of sulfur. The other organosulfur compounds found are also consistent with those found in the liquid phase by GC-SCD and have a relatively high vapor pressure. The remaining peaks are hydrocarbons, mainly cyclopentenes with different numbers of methyl groups. The peak around 3.5 min retention time, before methanethiol, could not be identified. Here, the aforementioned problem of the molecular masses of the substances being too low plays a role.

### Comparison of the Model Black Liquor with Real Black Liquor

**Figure 10:**
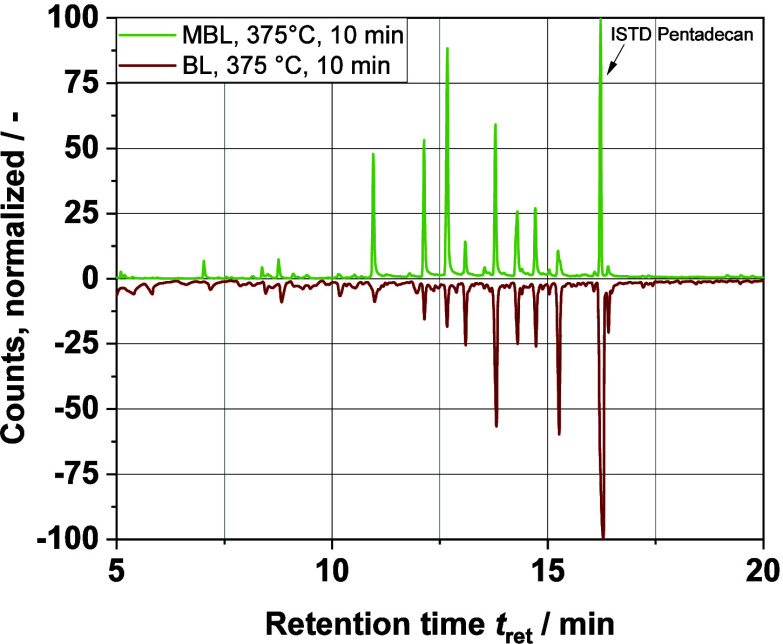
GC–MS chromatograms of MBL and real BL after HTL at *T*_R_ = 375 °C and *t*_R_ = 10 min. The chromatogram on the lower half is mirrored on the *X* axis.

As already shown in the previous section, the HS^–^ ions are clearly involved in the reactions during the HTL of the BL. To draw conclusions about the effects of HS^–^ on the depolymerization of the lignin, we investigated product yields and the change in the relative molecular mass at different salt concentrations in the feedstock. To adjust the concentration of HS^–^ ions as accurately as possible, we prepared model black liquors, as described in the Materials and Methods section. Figure 10 shows the GC–MS spectra of the extracted organic phase after HTL of a model liquor and the real BL at *T*_R_ = 375 °C and *t*_R_ = 10 min. It can be seen that the retention time of the peaks of the main components is the same for both analyzed samples. The main compounds produced are the same with the real BL and the MBL. One possible explanation for the different peak height could be the different composition of the organic content in the feedstock, i.e., the nonlignin organic compounds. Indeed, whereas in the MBL only the extracted lignin is used, the real BL also contains many other organic components, such as hemicellulose. Because both have approximately 9 wt % of biomass, the MBL has a higher lignin content than the BL. However, because our work is primarily concerned with the depolymerization of the lignin, this point can be neglected. In fact, the MBL even proved to be somewhat better with regard to the evaluation of the GC chromatograms: because of the absence of other organic components such as those from hemicellulose, there are fewer interfering small byproducts originating from the decomposition of the hemicellulose structures.

### Influence of HS^–^ Ions on the Gas Phase

**Figure 11:**
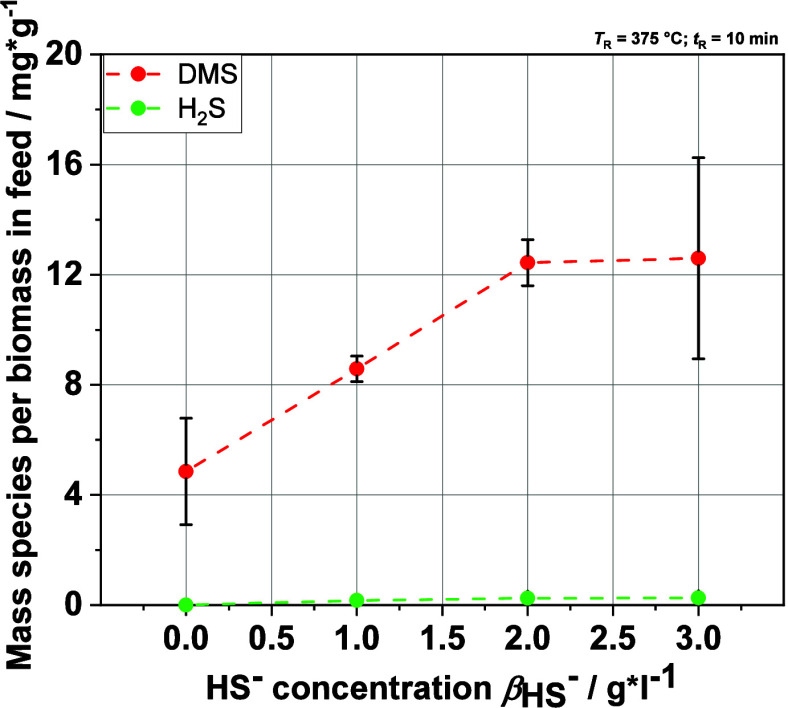
Evolution of the yields of DMS and H_2_S in regard to the biomass in the feed (lignin) in the analyzed product gas phase after HTL of MBLs via GC-FID.

The experiments with real BL show that DMS is one of the main organosulfur products. Therefore, looking at DMS production during the HTL of the MBL is a good way to observe the possible influences of the sulfide on the HTL process. In Figure 11, the increase of the DMS fraction with increasing HS^–^ concentration in the feed can be clearly seen. Thus, there is a clear link between the DMS production and the sulfide concentration in the feedstock, indicating that the mechanism by Karnofski et al. (see eqs 1–3) fits for the HTL process, too. Furthermore, the results also show that the HS^–^ ions remain in the liquid phase, as no significant increase in gaseous H_2_S was observed with increasing NaHS concentration. The pH value of the liquid product is decreasing only a little, which leads to the minor increase in the H_2_S yield with increasing sulfide concentration in the feed. Moreover, the gas analysis shows that DMS is mostly produced from lignin. Compared with the DMS yield from real BL at *T*_R_ = 375 °C and *t*_R_ = 20 min shown in Figure 8, the yield at 3 g·L^–1^ (*T*_R_ = 375 °C and *t*_R_ = 10 min) is at the same level at approximately 12 mg·g^–1^. These results fit together because not all of the biomass in the BL is lignin, and it can be expected that the experiment with the MBL with *t*_R_ = 20 min will lead to a higher DMS yield.

### Influence of HS^–^ Ions on the Depolymerization of Lignin

**Figure 12:**
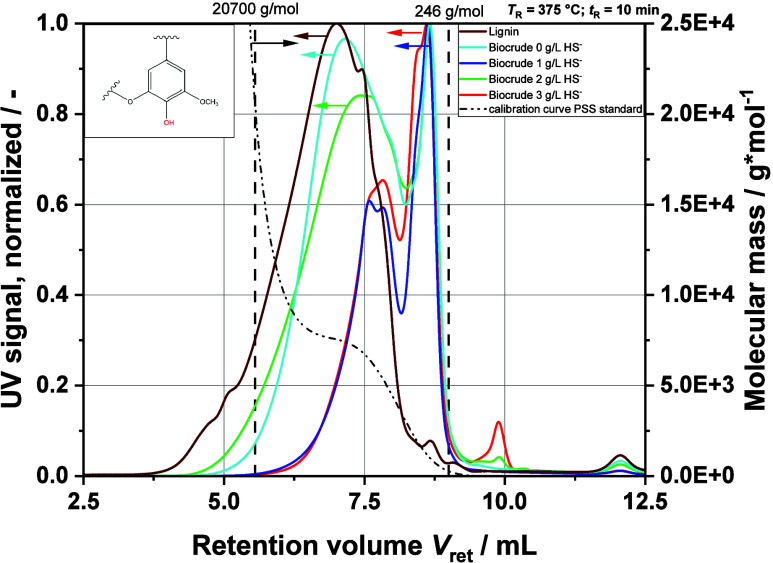
SEC chromatograms of the extracted organic product phase of the HTL of MBL together with the hardwood lignin used for the experiments. Calibrated ranges were from 246 to 20,700 g mol^–1^. Relative molecular mass decreases with a higher retention volume *V*_ret_.

The second step investigated the influence of the HS^–^ ion content on the organic phase in the liquid product with regard to molecular size. Figure 12 shows the recorded UV signals versus the retention volume *V*_ret_ detected with SEC analysis. With such a chromatogram, the lower the retention volume is, the higher is the relative molecular weight. These are relative values because absolute values are very difficult to obtain by SEC due to the lignin structure and without having appropriate standards for calibration of the method. Nevertheless, the observed trends and the evaluation of the effect of HS^–^ concentration on HTL are still meaningful. The calibrated range covers 246 to 20,700 g·mol^–1^. Four different chromatograms from extracted organic product of produced liquid phase at different temperatures are shown in Figure 12 (left *Y* axis). The hardwood lignin extracted from the feedstock is used as a reference. The calibration curve together with limits is included as well (right *Y* axis). The extraction of the organic phase was performed using the mixed liquid product phase obtained from three batch experiments (three repetitions). This was necessary to provide enough biocrude for the analyses. A clear trend is evident in the investigated concentration range of HS^–^ from 0 to 3 g*·*L^–1^ HS^–^ in the feedstock. The large peak at around 6000 g·mol^–1^ becomes smaller and shifts to a lower molecular weight. At 3 g*·*L^–1^ HS^–^, the peak is at about 4000 g·mol^–1^. Interestingly, the results for 1 and 2 g*·*L^–1^ HS^–^ do not fit optimally into the series. But all measurements show the formation of a molecular compound with a molecular weight of about 1500 g·mol^–1^, which, expressed in the number of syringyl groups (see aromatic structure in Figure 12; syringol, *M*_w_ = 154.16 g·mol^–1^), corresponds to approximately 10 aromatic rings. Clearly, HS^–^ contributes significantly to the depolymerization of lignin during HTL. This is surprising because the main drivers for depolymerization of lignin according to the literature are carbonates and hydroxides.^13^ Because of the higher reaction temperatures during HTL and a significantly higher pressure, it is possible that the reactions based on the Karnofski mechanism are accelerated compared to the Kraft process. Gierer et al. have explained a detailed mechanism for depolymerization of lignin with sulfides in their work.^40,52,53^ They describe how the HS^–^ ions force the cleavage of β-O-4 bonds, which are the most common type of bond in the lignin structure. With a higher HS^–^ concentration in the feed, it appears plausible that these cleavage reactions are favored.

**Figure 13:**
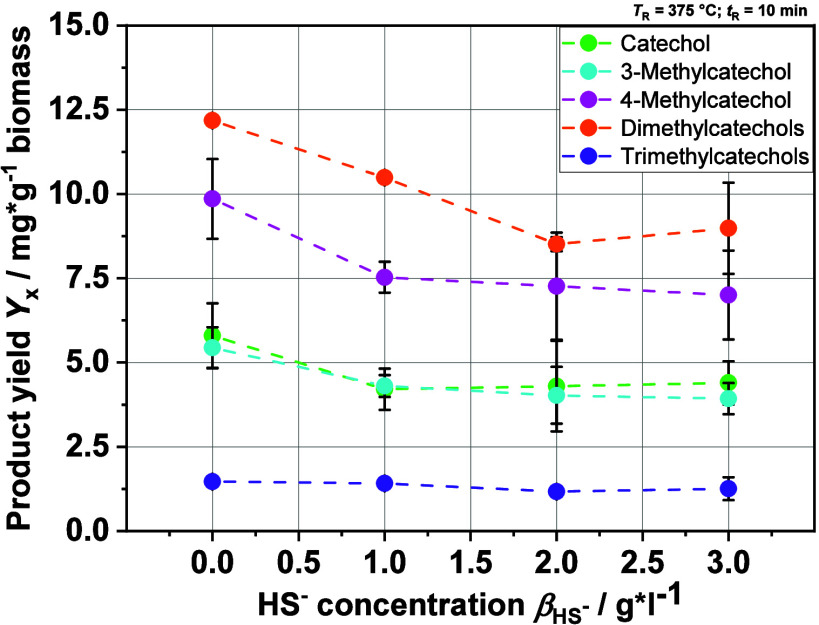
Monomer yields of different catechols in relation to the biomass in the feedstock (organic dry matter, here lignin). Dimethyl- and trimethylcatechols are quantified relatively by the peak area.

Finally, the monomer yields of various aromatics present in the liquid product phase were investigated (Figure 13). The figure focuses on the catechol and its methylated derivatives because these form the major part of the product spectrum. The influence of HS^–^ concentration on the yields of the various catechol and its derivates shows a general decreasing trend with increasing HS^–^ concentration, the largest decrease in yields is seen from 0 to 1 g·L^–1^ HS^–^, and the decrease is negligible in the range of 1–3 g·L^–1^ HS^–^. The amount of dissolved sulfide does not seem to be particularly relevant, which contradicts the SEC results showing an increased overall depolymerization. Based on these observations, we can hypothesize that HS^–^ ions, along with accelerating depolymerization, enhance the repolymerization of monomers, maybe by favoring the formation of free functional groups or radicals, which can react further with aromatic monomers. This could lead to the decrease shown in the yield of catechol and its derivates. Reactions between the hydroxy group of catechols and thiols or HS^–^ are highly unlikely because these are not thermodynamically favored.

Overall, it can be concluded that the HS^–^ concentration has a pronounced influence on the depolymerization of the lignin, leading mainly to compounds with a molecular weight of ca. 1500 g·mol^–1^, corresponding to ca. 10 aromatic rings. This accelerates with an increasing HS^–^ concentration. However, the main aromatic monomer products, the catechols and the methylated derivates, are barely affected. Therefore, the impact of HS^–^ on the HTL using BL is directly dependent on the goal of the process.

## Conclusions

Our study has shown that the influence of dissolved sulfides in the BL must be taken into account for the treatment of HTL of BL and downstream processes. The sulfur bound in inorganic molecules actively participates in the reaction pathways and is partly converted to organic sulfur compounds. The clearest indication of organic-bound sulfur is the production of DMS, which is detected in both the gas phase with the highest yield over 25 mg·g^–1^ biomass in feedstock and the liquid phase with a DMS concentration of up to 800 mg·L^–1^. Other organic sulfides as well as thiols were detected and partly quantified as well. A clear correlation between the HS^–^ concentration in the feedstock and the formation of DMS was found by using MBL with different HS^–^ concentrations as the HTL feedstock, which confirms the reaction path established by Karnofski et al. for the Kraft process. Other aspects such as the presence of methanethiol in the gas phase and the liquid phase also support this assumption. We were able to show that the HS^–^ concentration has an accelerating effect on lignin depolymerization reactions. Interestingly, the yields of the catechol and its derivates are only minimally affected by the addition of HS^–^ to the feedstock, and the effects of increasing HS^–^ concentration are negligible. Overall, this study provides new insights into the behavior of sulfur and especially the sulfide salts during the HTL of lignin by using BL directly, providing a good basis to evaluate the problems occurring from sulfur.

## Supporting Information Available

Weighed-in masses of the compounds and volume of the NaOH/KOH solution for each model black liquor (Table S1); volumes of BL used at specific reaction temperatures *T*_R_’s (Table S2); distribution coefficients for different catechols when using the described extraction procedure (Table S3); pH values for the liquid product after HTL of BL at three different reaction temperatures *T*_R_’s (300, 350, and 375 °C) and holding time *t*_R_ = 20 min (Figure S1); pH values for the liquid product after HTL of MBL at *T*_R_ = 375 °C and *t*_R_ = 10 min with different HS^–^ feedstock concentrations (Figure S2); sodium mass balance at different reaction temperatures *T*_R_’s (Figure S3); and potassium mass balance at different reaction temperatures *T*_R_’s (Figure S4) (PDF)

## Abbreviations

*T*_R_: reaction temperature
*t*_R_: holding time (batch)/residence time (cont.)
BL2F: black liquor to fuels
HTL: hydrothermal liquefaction
SEC: size exclusion chromatography
BL: black liquor
MBL: model black liquor
GC: gas chromatography
FID: flame ionization detector
TCD: temperature conductivity detector
SCD: sulfur chemiluminescence detector
ISTD: internal standard
*Y*_*x*_: product yield of compound *x*
HPLC: high-pressure liquid chromatography
MS: mass spectroscopy
IC(-An): ion chromatography (for anions)
ICP-OES: inductively coupled plasma-optical emission spectroscopy
DMS: dimethyl sulfide
DMDS: dimethyl disulfide
DMTS: dimethyl trisulfide
*w*_*x*_: mass fraction of species *x*
*M*_rel_: relative molecular mass
β_*x*_: mass concentration of species *x*
