# Supplementary material for: The Impact of Sulfur-Containing Inorganic Compounds during the Depolymerization of Lignin by Hydrothermal Liquefaction of Black Liquor
Source: Energy Fuels. 2024 Mar 19;38(7):6036–47. doi: 10.1021/acs.energyfuels.3c04737 (PMC11000222; doi:10.1021/acs.energyfuels.3c04737)
Supplement: Supplementary file 1 — ef3c04737_si_001.pdf [file ef3c04737_si_001.pdf]

Supporting Information

The Impact of Sulfur-containing Inorganic Compounds during the Depolymerization of Lignin by Hydrothermal Liquefaction of Black Liquor

Maximilian Wörner (maximilian.woerner@kit.edu)[*,†], Lukas Werner (lukaswerner94@web.de)[†], Ursel Hornung (ursel.hornung@kit.edu)[†], Nicholas Islongo Canabarro (nicholas.canabarro@sintef.no)[‡], David Baudouin (david.baudouin@psi.ch)[‡], Nicolaus Dahmen (nicolaus.dahmen@kit.edu)[†]*

[†]Karlsruhe Institute of Technology (KIT), Institute of Catalysis Research and Development (IKFT), Hermann-von-Helmholtz-Platz 1, 76344 Eggenstein-Leopoldshafen, Germany

[‡]Paul-Scherrer-Institute (PSI), Bioenergy and Catalysis Laboratory (LBK), Forschungsstrasse 111, 5232 Villigen, Switzerland

Table S1. Weighed-in masses of the compounds and volume of the NaOH/KOH solution for each model liquor

	**MBL-A**	**MBL-B**	**MBL-C**	**MBL-D**
**Na_2CO_3 / g**	1.777	1.778	1.778	1.781
**K_2CO_3 / g**	0.183	0.215	0.244	0.275
**Na_2SO_3 / g**	0.132	0.133	0.131	0.132
**Na_2SO_4 / g**	0.110	0.109	0.106	0.109
**$Na_2S_2O_3$ / g**	0.195	0.196	0.195	0.194
**Na_2S * 9 H_2O / g**	0	0.785	1.573	2.363
**Lignin / g**	9.001	8.998	8.998	9.002
**NaOH/KOH solution / ml**	8.75	7.5	6.25	5
**HS^- / $g{\cdot}l^{-1}$**	0	approx. 1	approx. 2	approx. 3

Table S2. Volumes of BL used at specific reaction temperatures T_R

T_R	250 – 275 ˚C	300 – 350 ˚C	375 ˚C	400 ˚C
V_{BL}	17,5 ml	15 ml	12,5 ml	5 ml

Table S3. Distribution coefficients for different catechols when using the described extraction procedure

Component name	Distribution coefficient K_i
Catechol	0.82
3-Methylcatechol	0.83
4-Methylcatechol	0.7

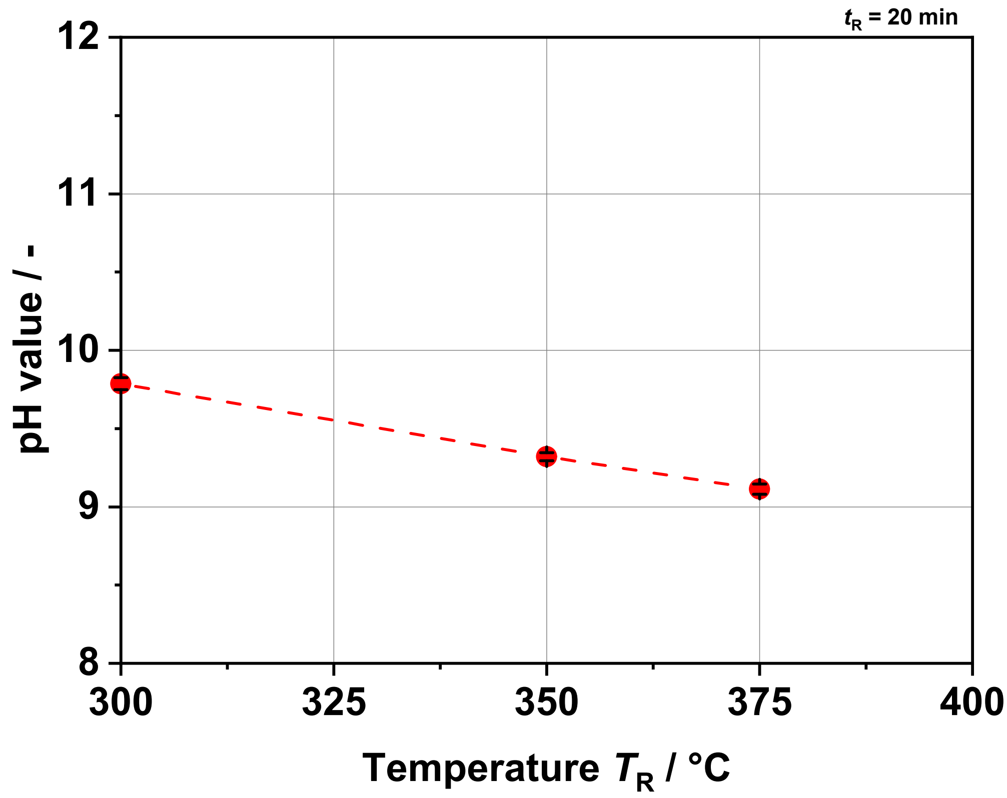

Figure S1. pH values for the liquid product after HTL of BL at three different reaction temperatures T_R (300 ˚C, 350 ˚C, 375 ˚C) and holding time t_R = 20 min

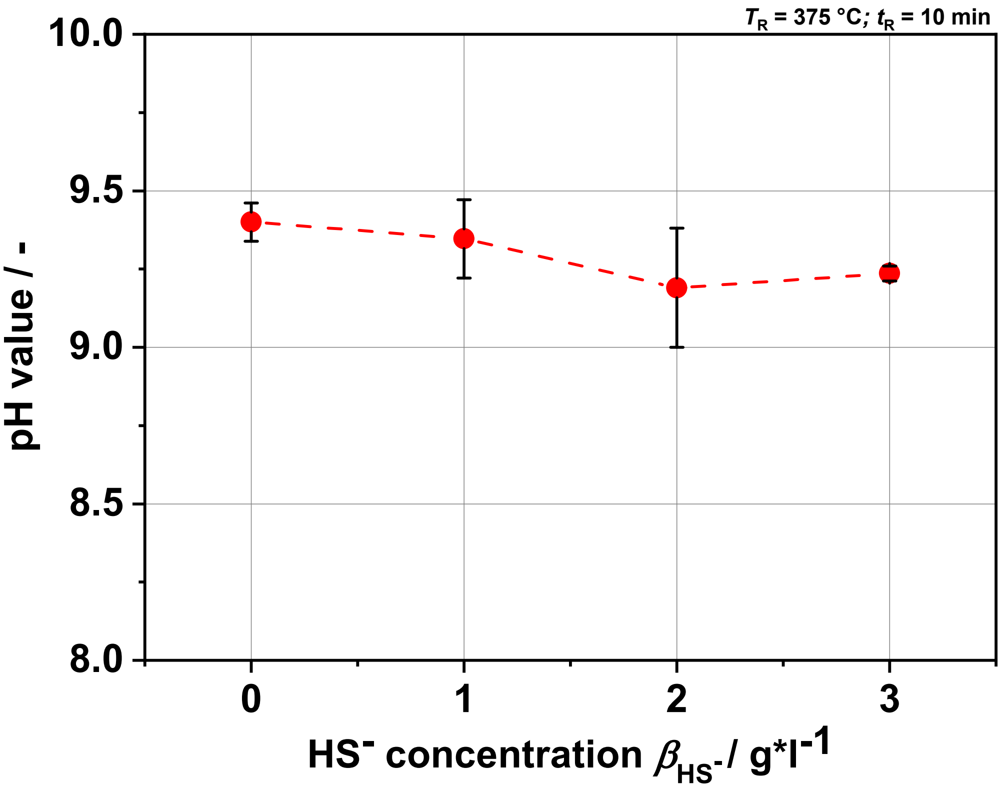

Figure S2. pH values for the liquid product after HTL of MBL at T_R = 375 ˚C and t_R = 10 min with different HS^- feedstock concentrations

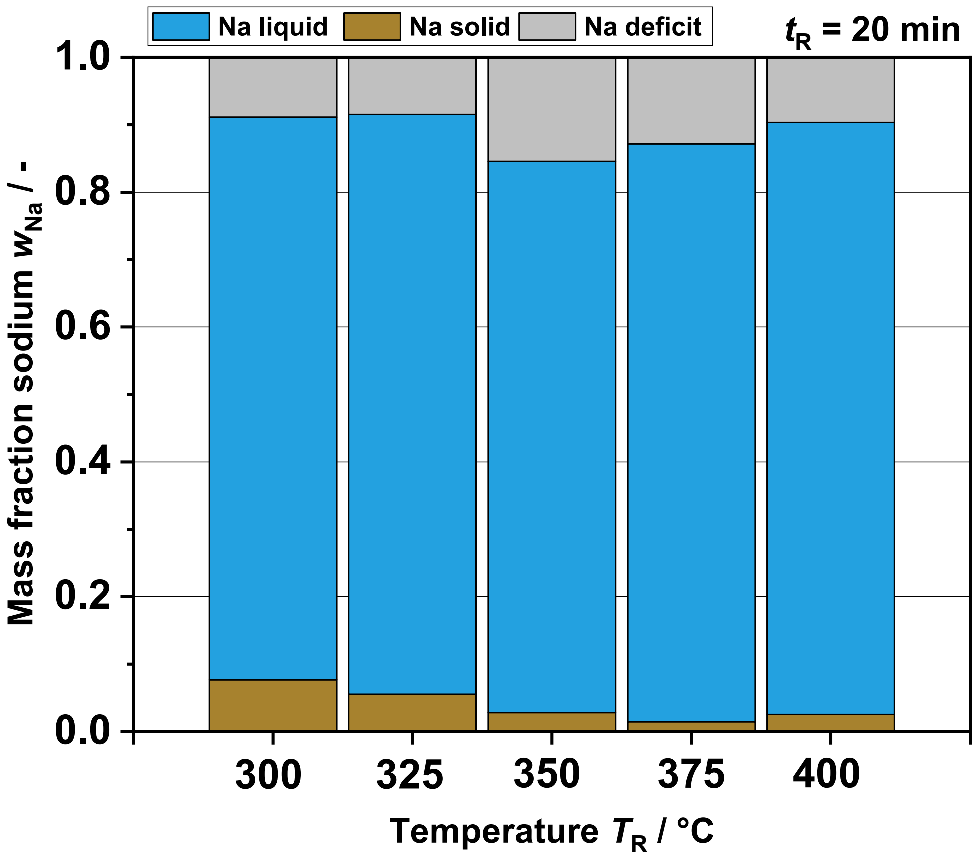

Figure S3. Sodium mass balance at different reaction temperatures T_R

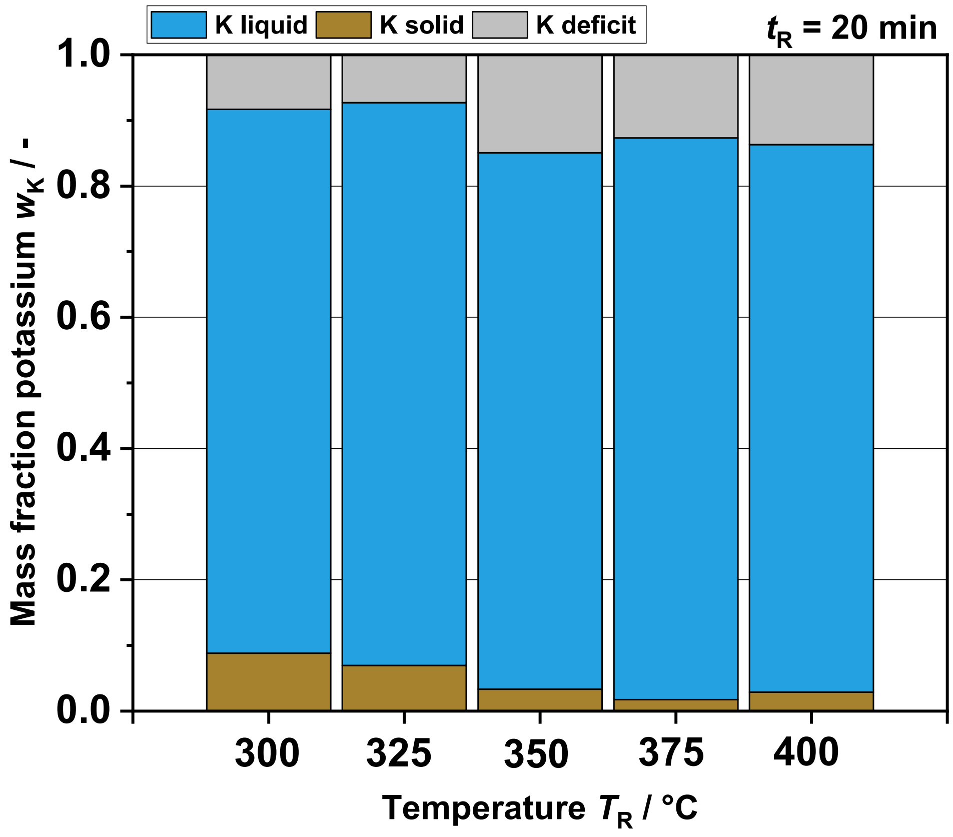

Figure S4. Potassium mass balance at different reaction temperatures T_R
